# Efficacy of Scalp Acupuncture in Patients With Post-stroke Hemiparesis: Meta-Analysis of Randomized Controlled Trials

**DOI:** 10.3389/fneur.2021.746567

**Published:** 2021-12-09

**Authors:** Yuan-Ju Huang, Chih-Shan Huang, Kuo-Feng Leng, Jia-Ying Sung, Sheng-Wei Cheng

**Affiliations:** ^1^Department of Neurology, Taipei Municipal Wan-Fang Hospital, Taipei Medical University, Taipei, Taiwan; ^2^Taipei Neuroscience Institute, Taipei Medical University, Taipei, Taiwan; ^3^Graduate Institute of Applied Statistics, College of Management, Fu Jen Catholic University, Taipei, Taiwan; ^4^Department of Neurology, School of Medicine, College of Medicine, Taipei Medical University, Taipei, Taiwan; ^5^Department of Nursing, Chang Gung University of Science and Technology, Taipei, Taiwan; ^6^Graduate Institute of Clinical Medicine, College of Medicine, Taipei Medical University, Taipei, Taiwan; ^7^Division of Gastroenterology, Department of Internal Medicine, Wan Fang Hospital, Taipei Medical University, Taipei, Taiwan

**Keywords:** stroke, meta-analysis, randomized controlled trial, scalp acupuncture, revised Cochrane risk of bias assessment

## Abstract

**Objectives:** To conduct a meta-analysis to assess the efficacy of scalp acupuncture (SA) in patients with stroke and consequent hemiparesis regardless of brain infarction or intracerebral hemorrhage.

**Methods:** A literature search of randomized controlled trials (RCTs) on SA for stroke was performed in five databases up to May 10, 2021. We investigated three types of outcome: motor function, sequelae of poststroke hemiparesis, and adverse effects. Methodological quality was assessed using the revised Cochrane risk of bias tool version 2.0.

**Results:** Of 1,063 papers, 30 RCTs involving Fugl–Meyer Assessment were selected, among which 10 and four RCTs were selected for evaluation of courses lasting of 1 and 3 months, respectively. The meta-analysis of 1- and 3-month courses revealed significant differences in the motor function of the SA plus Western standard treatment group vs. Western standard treatment only (medication plus rehabilitation; *P* < 0.001). A 3-month course tended to result in better outcomes than a 1-month course.

**Conclusions:** Our meta-analysis results reveal that SA improves motor function in patients with acute to chronic stroke, regardless of brain infarction or intracerebral hemorrhage. However, because of a lack of methodological quality, thoroughly planned clinical studies are still required.

## Introduction

Stroke is the sudden injury of neurons due to lack of blood supply to the brain, leading to the rapid development of a focal neurologic deficit. Globally, stroke is the second leading cause of death and a major cause of disability (1). It can be classified as ischemic (blood vessel occlusion) or hemorrhagic (blood vessel rupture). Common risk factors for ischemic and hemorrhagic stroke are age, race, sex, hypertension, diabetes, smoking, dyslipidemia, and alcohol use. For ischemic stroke, the specific risk factors are family history, atrial fibrillation, asymptomatic carotid stenosis, cardiac disease, sickle cell anemia, diet (high-sodium, low-potassium diet in overweight or elderly individuals), physical inactivity, obesity, hormone replacement therapy, hyperhomocysteinemia, hypercoagulability, lipoprotein(a), lipoprotein-associated phospholipase A2, inflammation, infection, and geography. For hemorrhagic stroke, the specific risk factors are antithrombolytic use, cerebral amyloid angiopathy, microbleeding, illicit drug use, dialysis, and tumors (2). Stroke incurs a considerable economic burden (3). Rehabilitation is a major part of stroke recovery (4). Furthermore, the use of sensory stimulation (transcutaneous electrical nerve stimulation or acupuncture) was reported to contribute to routine rehabilitation and improve poststroke hemiparesis (5).

Acupuncture has been a mainstream therapy in traditional and complementary medicine for >3,000 years for several diseases and also for poststroke recovery. Among the various methods, scalp acupuncture (SA) is a modern acupuncture technique integrating traditional Chinese needling methods with Western medical knowledge of representative areas of the cerebral cortex. SA is performed based on the functionality of brain areas to stimulate different scalp zones with needles; such stimulation improves the reflexivity of certain nerves and is mostly applied in individuals with acute and chronic central nervous system disorders, especially stroke (6). The mechanisms that are currently considered possible for SA include reducing brain edema, diminishing cerebral vessel permeability, promoting reparation of blood–brain barrier damage, decreasing inflammation, improving energy metabolism, and relieving the inhibitive generalization of the whole brain neuron function (7).

In the past decade, several systematic reviews and meta-analyses have been published that have assessed the effect of SA on acute hypertensive intracerebral hemorrhage (8) and postapoplectic aphasia (9). In addition, a meta-analysis of animal studies reported promising results, finding that SA improved infarct volume and neurological function score (10). Moreover, in Young-Nim et al. (11) obtained similar results in their meta-analysis; however, the intervention methods were mixed (SA and body acupuncture). Therefore, we decided to investigate whether additional SA has benefits for patients with stroke. The purpose of this study was to comprehensively review randomized controlled trials (RCTs) that evaluated the effectiveness of SA for motor dysfunction in stroke. We wanted to obtain a clinically meaningful synthesis of the existing evidence to determine whether SA may be a suitable complementary therapy for the motor sequelae of stroke.

## Materials and Methods

### Types of Studies

Randomized controlled trials (RCTs) evaluating the effect of SA on motor function in stroke with the control group receiving modern standard treatment or conventional treatment were included in this study.

### Types of Participants

Randomized controlled trials (RCTs) that included patients of any age or sex with acute or chronic stroke were eligible. SA was administered to patients with stroke as diagnosed through CT or MRI. The control group in these RCTs received only standard Western treatment for stroke in a neurological ward.

### Types of Intervention

Randomized controlled trials (RCTs) were included regardless of treatment duration and number of sessions, but acupoint selection was limited to the scalp; ears and body acupoints were excluded. The control group patients received Western standard treatment (medications and rehabilitation). Patients in the trial groups received SA therapy (SA of standard international acupuncture nomenclature) in addition to the same standard treatment as that in the control groups. Concerning SA, acupoints are mostly in the motor areas (the anterior oblique line of the vertex-temporal, MS 6) and sensory areas (the posterior oblique line of the vertex-temporal, MS 7) of the contralateral scalp of hemiplegic limbs. The needle retention time was mostly between 0.5 and 2 h, and the depth of needle insertion was 2–4 cm. The frequency was 5–7 days per week and once a day. The treatment courses ranged from 2 weeks to 6 months. Those who performed SA in these studies were experienced doctors.

### Outcomes Measured

We investigated three types of outcome: motor function, sequelae of poststroke hemiparesis, and adverse effects. We evaluated motor function in the upper and lower extremities at the end of the first and third months. The assessment was performed using the Fugl–Meyer Assessment (FMA), a stroke-specific, performance-based impairment index (12, 13). The FMA motor score ranges from 0 (hemiplegia) to 100 (normal motor performance) points, which were divided into 66 points for the upper extremity and 34 points for the lower extremity.

### Literature Search

We searched databases, namely, PubMed, Embase, the Cochrane library, Airiti Library, and China National Knowledge Infrastructure (until May 2021) by two reviewers (YJH and CSH). The keywords used for the search were “scalp acupuncture” and “stroke.” The type of article searched for was the RCT. We focused on poststroke hemiparesis or hemiplegia and excluded unwanted outcomes (poststroke dysarthria, poststroke dysphagia, poststroke aphasia, central poststroke pain, poststroke depression, poststroke cognitive impairment, etc.). The reference lists of all relevant articles were searched for further studies. There was no language limitation.

### Data Extraction and Quality Assessment

Two reviewers (YJH and CSH) independently assessed all studies and independently extracted eligible data from the trial reports by using a data extraction form; data were cross-checked for accuracy before use. Disagreement was resolved through discussion with a third reviewer (SWC) if necessary. The authors of the trials were contacted and requested to provide missing data.

Quality assessment was conducted by revised Cochrane risk of bias tool 2.0 (RoB2.0) to determine whether the trials had the following concerns regarding internal validity: 1) risk of bias arising from the randomization process; 2) risk of bias due to deviation from intended intervention; 3) missing outcome data; 4) risk of bias in outcome measurements; or 5) risk of bias in the selection of the reported results. We conducted the risk of bias of summary and graph by using Review Manager 5.4.

### Data Analysis

Heterogeneity between trial results was tested using the standard I^2^ test. Results are reported as odds ratios with corresponding 95% CIs for dichotomous data. If continuous data were available, the weighted mean difference or standardized mean difference was calculated. Statistical analysis was performed using the statistical software R version 3.6.0 and the meta package.

## Results

### Search Strategy and General Information

**Figure 1:**
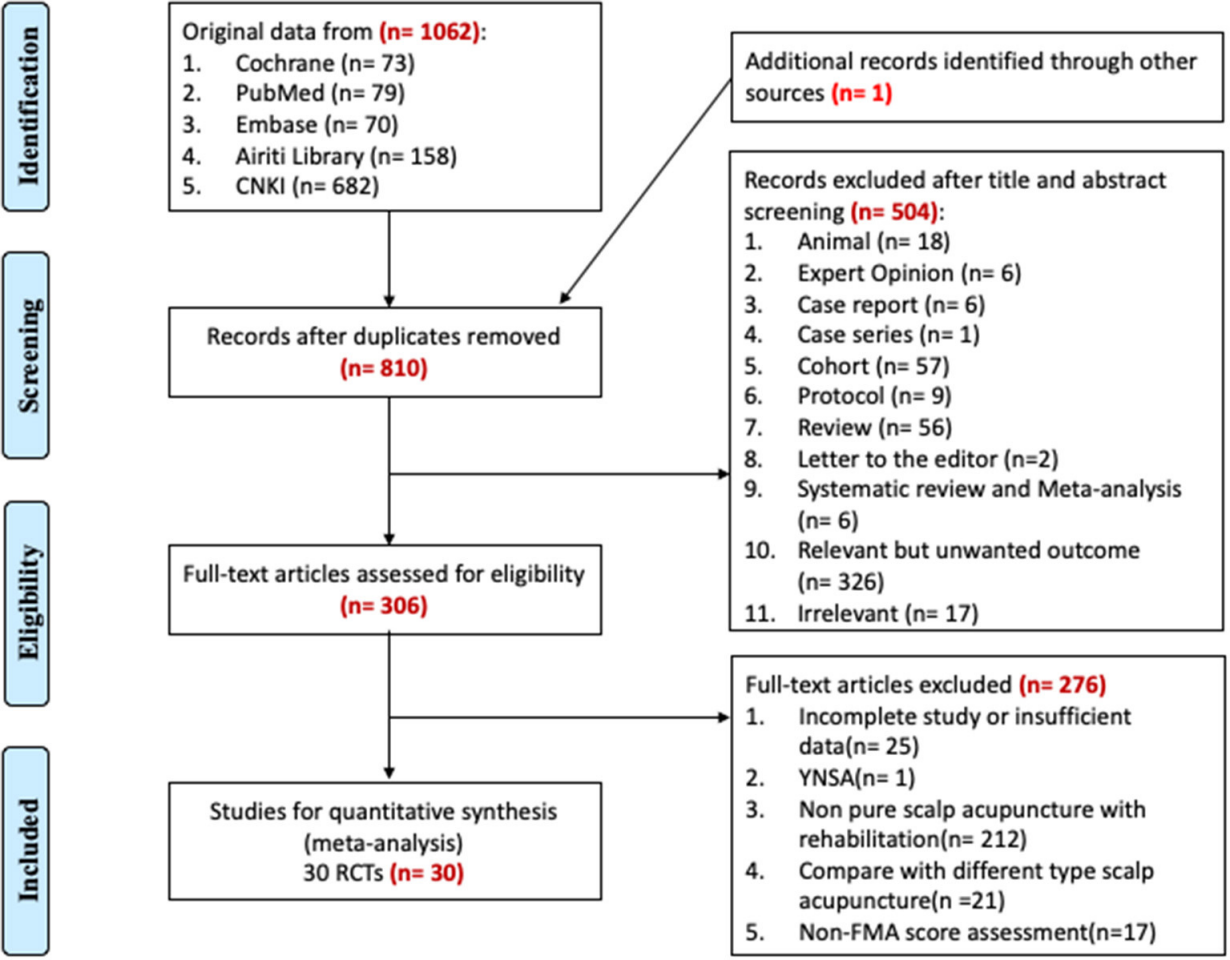
Flow diagram of the literature search.

Based on the search strategy, we manually retrieved 1,062 papers found in the five databases and one from another source (manual search using Google Scholar). These studies were published from 1975 to 2021, and 253 papers had duplicate titles. Of the remaining 810 papers, after screening the abstracts, 18 animal studies, six expert opinions, seven case studies, 57 cohort studies, nine protocols, six systematic reviews or meta-analyses, 56 narrative reviews, 326 relevant articles with an unwanted outcome (poststroke dysarthria, poststroke dysphagia, poststroke aphasia, central poststroke pain, poststroke depression, poststroke cognitive impairment, etc.), and 17 irrelevant articles were excluded; the remaining papers with full text available were selected. Among the 306 RCTs selected, 25 papers with incomplete or insufficient data, one paper regarding noninternational-standard SA (Yamamoto new SA), 212 papers regarding impure SA with rehabilitation, 21 papers comparing different SA types, and 17 papers not including FMA scores were excluded. A total of 30 RCT papers containing FMA scores were finally selected (14–43). The process used for the literature search for the application in the systematic review is summarized in Figure 1.

All these 30 RCTs were conducted in China, and the corresponding articles were published from 2007 to 2019 (Table 1). Of these RCTs, 15 mentioned 1-month results; five mentioned 6-week results; six mentioned 2-month results; and six mentioned 3-month results. We assessed both upper and lower extremity strengths, so we selected only papers with complete FMA scores (including the upper and lower extremities with a total score of 100). We selected treatment courses of 1 and 3 months and then conducted the meta-analysis. A total of 1,043 patients with acute or chronic stroke from 12 reports were included.

**Table 1:** Characteristics of the included RCTs.

**Study No**.	**Author**	**Year**	**Journal**	**Age(years)**		**Sex (male/female)**		**Stroke type (infarction/hemorrhage)**		**Course of treatment**	**Outcomes FMA U: upper; L: lower (*P*-value); S: sequelae; A: adverse effect**
				**experimental**	**control**	**experimental**	**control**	**experimental**	**control**		
1	Xie	2007	Chinese Journal of Rehabilitation Theory and Practice	53 ± 9.3	56.5 ± 6.4	24/17	22/17	41	39	3 months	FMA:U+L (*P* < 0.05)
2	Li	2009	Shanghai J Acu-mox	61 ± 6	63 ± 6	24/21	27/18	34/11	35/10	6 weeks	FMA:U+L (*P* < 0.01), S
3	Ma	2010	Chinese Journal of Rehabilitation	62.1 ± 0.5	61.4 ± 0.2	8/7	9/6	15/0	15/0	3 weeks	FMA:U+L (*P* < 0.05)
4	Fu	2011	Chinese Journal of Traditional Medical Science and Technology	38-81^*^		37/27^*^		36/28^*^		2 months	FMA:U+L (*P* < 0.01)
5	Qin	2013	Journal of Clinical Acupuncture and Moxibustion	59.95 ± 6.35	60.95 ± 7.12	12/8	11/9	20	20	3 months	FMA:L (*P* < 0.05)
6	Kong	2014	Practical Journal of Medicine and Pharmacy	60 ± 9.72	87 ± 9.72	23/10	21/12	33	33	3 months	FMA:U (*P* < 0.01)
7	Zhang	2015	Chinese Medicine Modern Distance Education of China	63 ± 4	62 ± 5	16/14	15/15	18/12	17/13	1 month	FMA:U (*P* < 0.05)
8	Xu	2015	Hebei Medical Journal	58.3 ± 11.2	61.7 ± 9.1	45/15	42/18	48/12	44/16	1 month	FMA:U+L (*P* < 0.05)
9	Qin	2015	Chinese Journal of Gerontology	64.7 ± 5.66	65.3 ± 7.87	39/19	36/22	58	58	3 months	FMA:U+L (*P* < 0.05)
10	Tan	2015	Chinese Journal of Integrated Traditional and Western Medicine	58.4 ± 10.5	59.3 ± 11.5	40/40	42/38	60	60	1 month	FMA:U (*P* < 0.01)
11	Liu	2016	Modern Journal of Integrated Traditional Chinese and Western Medicine	58.6 ± 4.9	60.5 ± 3.9	17/13	20/10	23/7	20/10	1 month	FMA:L (*P* < 0.05)
12	Dou	2016	World Latest Medicine Information	58.7 ± 2.35	59.8 ± 2.77	23/10	22/11	33	33	1 month	FMA:U+L (*P* < 0.05)
13	Chen	2016	Practical Clinical Journal of Integrated Traditional Chinese and Western Medicine	56.1 ± 6.45	55.29 ± 5.86	18/11	16/14	29/0	30/0	1 month	FMA:U+L (*P* < 0.01)
14	Wang	2017	Chinese acupuncture and moxibustion	62 ± 10	63 ± 8	42/18	50/10	21/39	26/34	1 month	FMA:U+L (*P* < 0.05)
15	Yang	2017	China Health Standard Management	47 ± 8.4	48.4 ± 2.5	10/8	11/7	12/6	9/9	1 month	FMA:U+L (*P* < 0.05)
16	Yin	2017	China Health Standard Management	65.2 ± 3.4	64.5 ± 3.2	28/22	26/24	37/13	38/12	2 months	FMA:U (*P* < 0.05)
17	Pan	2017	Information on Traditional Chinese Medicine	64.12 ± 9.69	62.91 ± 10.82	29/24	28/25	53	53	1 month	FMA:U (*P* < 0.05), S
18	Xu	2018	Chinese Medicine Modern Distance Education of China	58.44 ± 8.26	58.28 ± 7.95	12/13	13/12	25	25	1 month	FMA:U+L (*P* < 0.01)
19	Xiao	2018	Jilin Journal of Chinese Medicine	57.14 ± 9.67^*^		63/45^*^		54/0	54/0	3 months	FMA:U+L (*P* < 0.05)
20	Hu	2018	Asia-Pacific Traditional Medicine	64.7 ± 6.1^*^		61/51^*^		73/39^*^		2 months	FMA:U+L (*P* < 0.05)
21	Sun	2019	Chinese Journal of Convalescent Medicine	63.26 ± 2.56	63.15 ± 2.15	23/22	22/23	45/0	45/0	6 months	FMA:U (*P* < 0.05), A
22	Zhang	2019	Clinical Education of General Practice	54.49 ± 4.35	52.44 ± 12.13	23/17	21/19	29/11	26/14	6 weeks	FMA:U (*P* < 0.05)
23	Zhang	2019	Chinese Journal of Gerontology	64.3 ± 9.2	64 ± 9.6	18/16	17/17	20/14	20/14	3 months	FMA:U+L (*P* < 0.05)
24	Zhu	2019	Chinese Journal of Rehabilitation Medicine	49 ± 3.7	54 ± 1.9	27/13	17/23	40	40	2 months	FMA:L (*P* < 0.01)
25	Li	2019	China Modern Medicine	67.96 ± 7.98	68.65 ± 8.25	22/16	23/17	38/0	40/0	6 weeks	FMA:U (*P* < 0.05)
26	Hu	2019	Shanghai J Acu-mox	62 ± 10	62 ± 8	19/15	17/17	23/8/3^**^	26/6/2^**^	1 month	FMA:U+L (*P* < 0.01)
27	Ye	2019	New Journal of Traditional Chinese Medicine	65.12 ± 7.31	65.03 ± 7.12	33/13	34/12	46/0	46/0	2 months	FMA:U+L (*P* < 0.01)
28	Zhao	2019	Jilin Journal of Chinese Medicine	60.76 ± 7.43	59.89 ± 8.57	27/18	29/16	45	45	2 weeks	FMA:U+L (*P* < 0.05)
29	Chen	2019	Journal of Preventive Medicine of Chinese People's Liberation Army	62.53 ± 11.71	68.63 ± 4.79	12/8	11/9	20	20	6 weeks	FMA:U+L (*P* < 0.05)
30	Ma	2019	New Journal of Traditional Chinese Medicine	67.2 ± 5.7	65.4 ± 4.8	10/10	9/11	20	20	1 month	FMA:U (*P* < 0.05)

* *The data was provided by the author before randomization*.

** *Mixed type stroke*.

### Quality of the Trials

**Figure 2:**
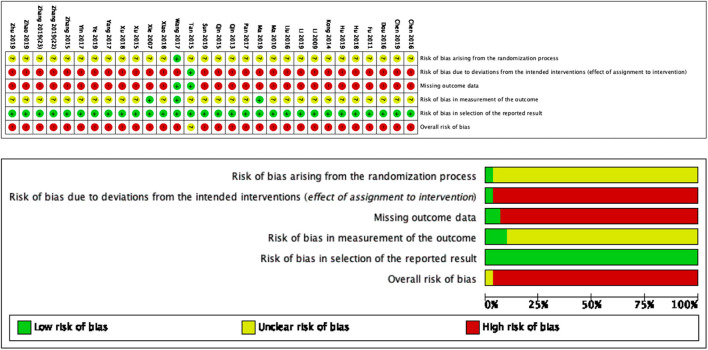
Risk of bias summary and graph for each risk of bias item presented as percentages across all included studies.

Based on Figure 2, we determined the overall quality of the included studies to be low by RoB2.0. For the first domain (risk of bias arising from the randomization process), the generally unclear risk was indicated. All trials used a randomization method, including a random number generator (calculator) and a sequencing method. One trial (28) described a grade-A level of adequate concealment of randomization in which the patients were allocated according to calculated random numbers sealed in opaque envelopes. For the second domain (risk of bias due to deviation from the intended intervention), the risk was generally high because only one trial (21) included a control group for which sham acupuncture was implemented. The remaining 29 trials did not mention blinding or intention-to-treat analysis. For the third domain (missing outcome data), the risk was generally high because only two trials (21, 28) recorded missing data. For the fourth domain (risk of bias in outcome measurement), the risk was generally unclear because only three trials (14, 28, 37) mentioned that the assessor did not participate in the therapy. For the fifth domain (risk of bias in the selection of the reported results), the risk was generally low. All trials conducted a comparative analysis between the SA group and control group.

### Death or Dependency

None of the trials used death or dependency as a primary outcome measure.

### Muscle Strength Improvement (FMA)

**Figure 3:**
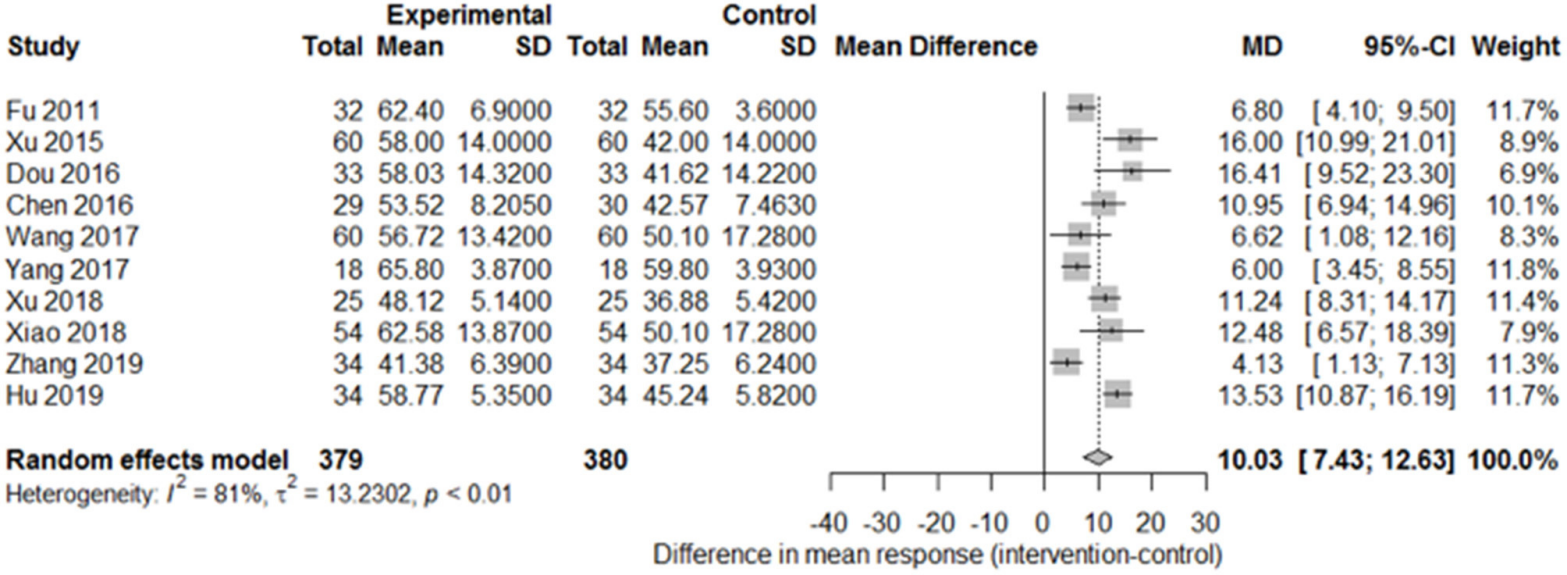
Forest plot of the comparison of mean difference in hemiparesis improvement after treatment with SA vs. control after a 1-month course.

**Figure 4:**
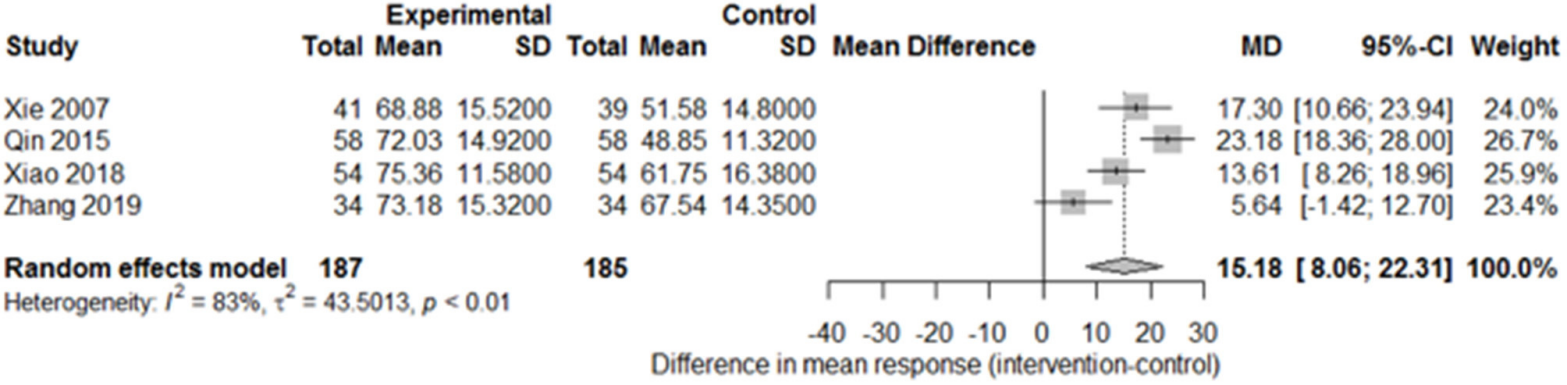
Forest plot of the comparison of mean difference in hemiparesis improvement after treatment with SA vs. control after a 3-month course.

**Figure 5:**
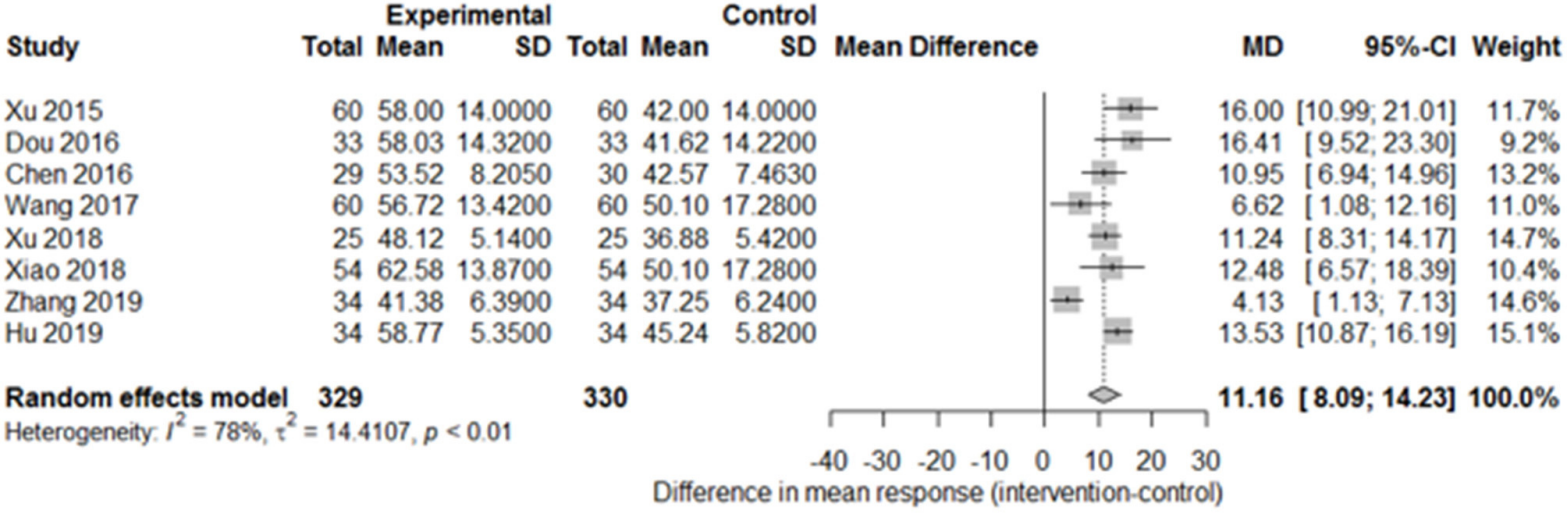
Forest plot of the comparison of mean difference in hemiparesis improvement after treatment with SA vs. control after a 1-month course and removal of two papers on special rehabilitation for the sensitivity test.

Among the papers on 30 independent trials, 10 reporting the complete FMA score described the 1-month outcome (Figure 3; mean difference, 10.3 [95% CI, 7.43–12.63]; *P* < 0.01), and four reporting the complete FMA score described the 3-month outcome (Figure 4; mean difference, 15.18 [95% CI, 8.06–22.31]; *P* < 0.01). Two papers (17, 29) with a 1-month course mentioned that special rehabilitation may diminish the effect of SA; therefore, we removed these papers and reconducted a sensitivity test of the meta-analysis (Figure 5; mean difference, 11.16 [95% CI, 8.09–14.23]; *P* < 0.01). There were no homogeneity in the consistency of the trial results (1 month: I^2^ = 78% and 3 months: I^2^ = 83%). SA was found to have significantly improved hemiparesis, as represented using the FMA score and when compared with the control group. The outcome after 3 months of SA (mean difference in FMA score: 15.18) seemed to be better than that after 1 month of SA (mean difference in FMA score: 11.16).

### Sequelae of Hemiparesis-Associated Symptoms

**Figure 6:**
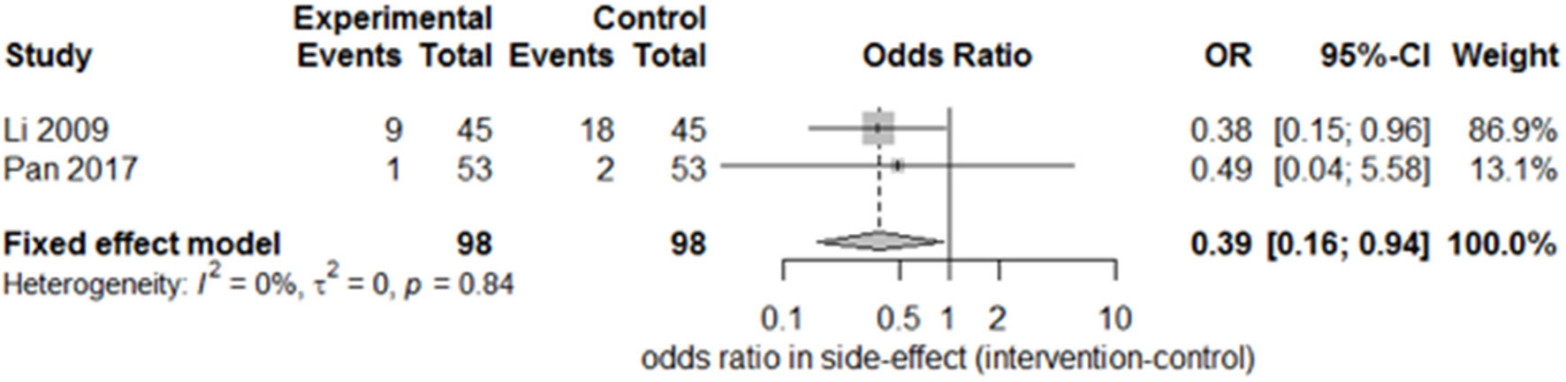
Forest plot of the odds ratio comparison for shoulder–hand syndrome in the SA group vs. the control group.

**Figure 7:**
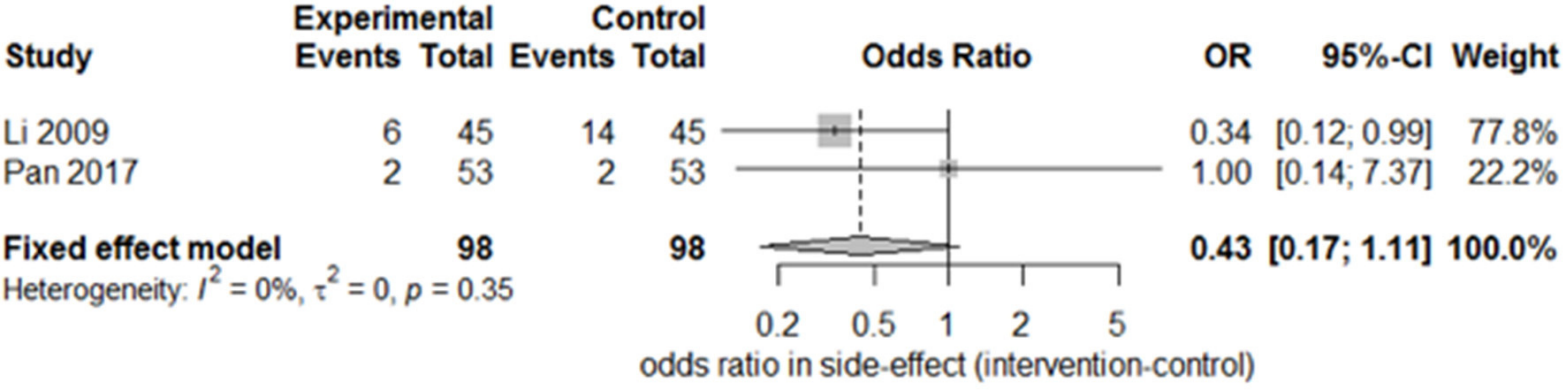
Forest plot of odds ratio comparison for shoulder pain in the SA group vs. the control group.

**Figure 8:**
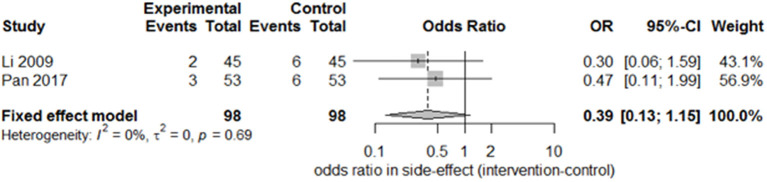
Forest plot of the odds ratio comparison for muscle atrophy in the SA group vs. the control group.

Only two studies (15, 27) recorded the sequelae of hemiparesis, such as shoulder–hand syndrome, shoulder pain, and muscle atrophy. Only one study (15) recorded complete muscle spasticity and bedsores. After pooling the results of the two papers, we found a significant difference in the incidence of shoulder–hand syndrome (Figure 6; odds ratio, 0.39 [95% CI, 0.16–0.94]) between the experimental and control groups, but no difference in that of shoulder pain (Figure 7; odds ratio, 0.43 [95% CI, 0.17–1.11]) or muscle atrophy (Figure 8; odds ratio, 0.39 [95% CI, 0.13–1.15]).

### Adverse Events

Adverse events were mentioned in one trial (38). In the SA groups, the abnormal response rate for dizziness and local skin redness was 6.67% (3/45), whereas it was 8.89% (4/45) in the control group. The difference was not significant.

### Publication Bias

**Figure 9:**
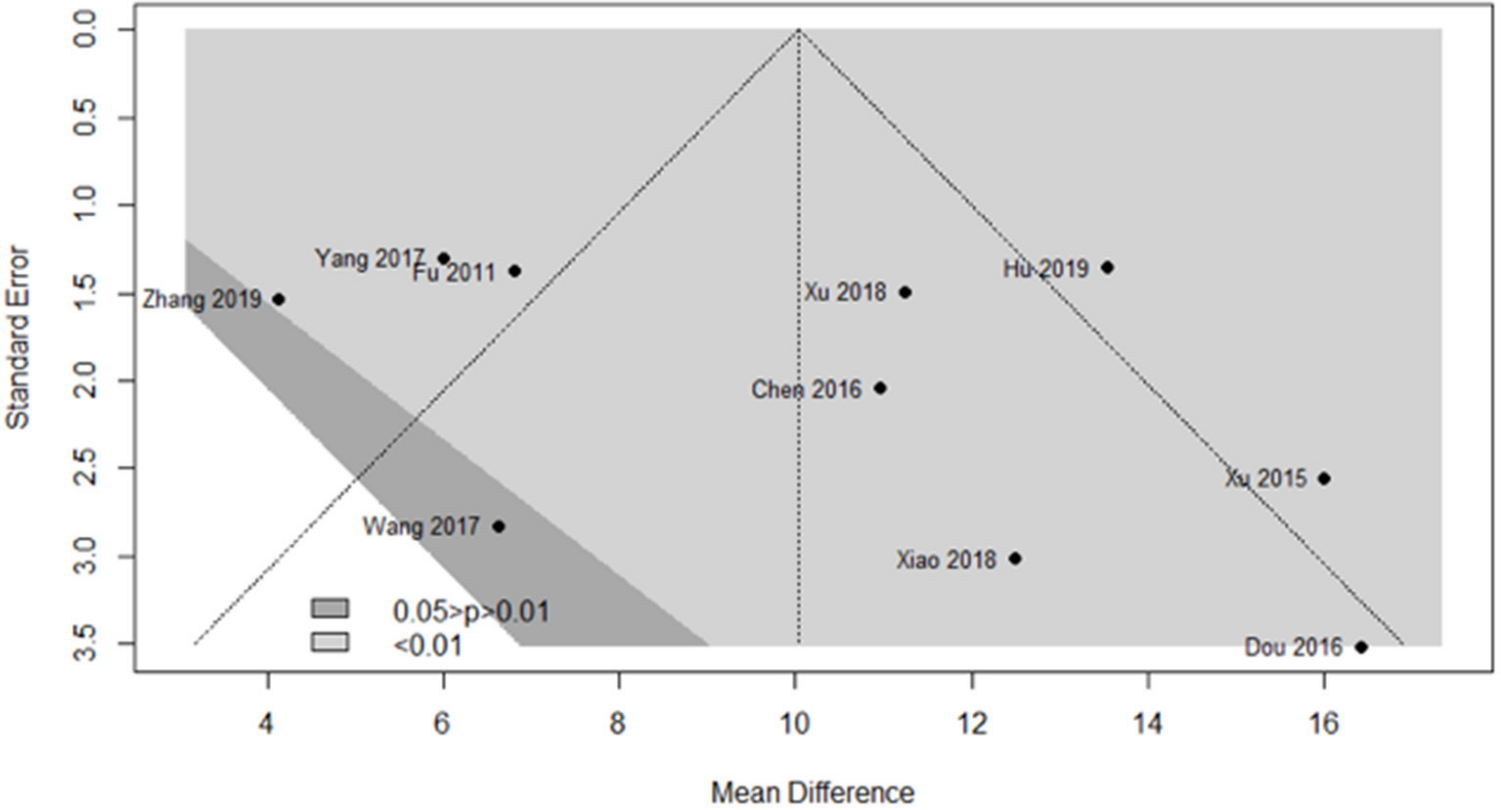
Funnel plot of the clinical effects in the SA group vs. the control group after a 1-month course.

We used a contour-enhanced funnel plot to differentiate between asymmetry due to publication bias and other reasons. Figure 9 demonstrates that almost all trials obtained a significant result favoring the experimental group, with *P* < 0.01 (light gray region) in nine trials and 0.01 < *P* < 0.05 (dark gray region) in one trial. However, publication bias was noted in five trials from the funnel plot.

## Discussion

This is the first meta-analysis to specifically evaluate the clinical outcomes of SA for poststroke hemiparesis, regardless of hemorrhage or infarction. The results indicate that SA is effective for improving motor dysfunction in patients with stroke.

Other clinical studies have suggested that SA therapy may be a suitable complementary treatment for poststroke neurological dysfunctions, namely, dysphagia (44), aphasia (45), central poststroke pain (46), depression (47), and cognitive impairment (48). In this meta-analysis, after searching five databases up to May 2021, 30 RCTs with FMA scores were selected; a systematic review of the clinical research on SA for stroke was conducted, and each study was qualitatively assessed. The disease period investigated in the meta-analysis varied from acute to chronic. We wished to focus only on motor function; therefore, we used the FMA score rather than the National Institutes of Health Stroke Score or Barthel index. Among the 30 RCT papers, 10 described the outcome after 1 month and 4 mentioned the outcome after 3 months. For the sensitivity test, two papers were removed from the 1-month course because of special rehabilitation, which may have influenced the effect of SA therapy. We observed that Zhang (40) reported markedly lower efficacy of SA therapy in contrast to other papers reporting 1- or 3-month courses; we speculated that this was related to the different areas of the scalp acupoints used, which were balance areas (MS 14) rather than the motor and sensory areas. When combined with Western standard medicine (medication and rehabilitation), SA obtained significantly better results in the meta-analysis on hemiparesis improvement during the stroke recovery period. A 3-month course tended to be better than a 1-month course. Regarding sequelae of hemiparesis-associated symptoms—namely, shoulder–hand syndrome, shoulder pain, and muscle atrophy—we observed additional SA benefits, although the difference was significant only in the incidence of shoulder–hand syndrome and not in the incidence of shoulder pain or muscle atrophy due to the small sample.

Almost all of the RCTs included in this meta-analysis were conducted without blinding due to the limitation inherent in the acupuncture procedure; thus, confounding factors, such as the placebo effect, may have been present. Only one paper (21) mentioned sham acupuncture as a control. Only two papers (21, 28) reported dropouts, and one paper (38) reported an adverse effect of SA; most papers did not mention these factors, which resulted in low assessment quality when the revised Cochrane risk of bias tool 2.0 was used.

There was no consistency in the rehabilitation method used in the studies in this meta-analysis, which may have affected the results on SA accuracy and efficacy. In most RCTs, physical therapy and occupational therapy were employed as rehabilitation interventions. One RCT added visual scanning and sensory integration training to the rehabilitation (17). Another study used special rehabilitation—constraint-induced movement therapy (29). The different rehabilitation types in these two studies weakened the effect of SA, which indicated that special rehabilitation may have a role in the improvement of poststroke hemiparesis. Different types of rehabilitation with integrated SA therapy as complementary treatment could be used in patients with stroke.

Not all RCTs compared the effect of SA between brain infarction and hemorrhage. The populations in these RCTs were all Asian, and there is a lack of data on Western populations; therefore, whether racial differences affect the outcome of SA must be determined in the future.

Only one study (38) reported the 6-month outcome of SA; significant improvement in poststroke hemiparesis was discovered, which indicated that longer SA duration may result in added benefits in patients with stroke. Another trial (49) assessed the degree of improvement of limb dysfunction by noting the scalp needle retention time in patients with stroke. The scalp needles in the treatment group were indwelled for 7–10 h and in the control group for 30 min. The results indicated greater improvement in limb dysfunction and activities of daily living after stroke when scalp needle retention was longer. The duration effect of SA therapy warrants further research.

## Data Availability Statement

The original contributions presented in the study are included in the article/supplementary material, further inquiries can be directed to the corresponding author/s.

## Ethics Statement

Ethical review and approval was not required for the study on human participants in accordance with the local legislation and institutional requirements. Written informed consent for participation was not required for this study in accordance with the national legislation and the institutional requirements.

## Author Contributions

Category 1: Y-JH conception and design of study and acquisition of data. Y-JH, C-SH, K-FL, J-YS, and S-WC analysis and/or interpretation of data. Category 2: Y-JH drafting the manuscript. Category 3: C-SH, K-FL, J-YS, and S-WC revising the manuscript critically for important intellectual content. Y-JH, C-SH, K-FL, J-YS, and S-WC approval of the version of the manuscript to be published. All authors contributed to the article and approved the submitted version.

## Conflict of Interest

The authors declare that the research was conducted in the absence of any commercial or financial relationships that could be construed as a potential conflict of interest.

## Publisher's Note

All claims expressed in this article are solely those of the authors and do not necessarily represent those of their affiliated organizations, or those of the publisher, the editors and the reviewers. Any product that may be evaluated in this article, or claim that may be made by its manufacturer, is not guaranteed or endorsed by the publisher.
